# Quality of life in monogenic diabetes (a case report)

**DOI:** 10.1186/1687-9856-2013-S1-P25

**Published:** 2013-10-03

**Authors:** Nur Rochmah, Muhammad Faizi, Ahmad Yuniari, EP Netty

**Affiliations:** 1 Department of Child Health, School of Medicine, Airlangga University-Dr Soetomo Hospital, Surabaya, Indonesia

## 

Monogenic diabetes in children mostly results from mutations in genes that regulate beta-cell function. It may be dominantly or recessively inherited or may be a de novo mutation and hence a spontaneous case. Quality of life is important in management of patient with monogenic diabetes. Objective is to analyze quality of life in a patient with monogenic diabetes.

A-19year old, girl, 29.5 kg, sufferred from polyuria, polydipsia, and polyphagia. Family history of diabetes was positive. Physical examination revealed non obese, with colateral vein, and hepatosplenomegaly. Laboratory examination revealed fasting blood glucose 275 mg/dL, hemoglobin A_1C_/A1C 10.3%, C-peptide 4.2ng/mL (normal:0.9-7.1ng/mL), ALT:110 U/L, AST:115 U/L; HDL:70 µg/dL, LDL:57mg/dL, total cholesterol:319mg/dL, and triglyceride:2030 mg/dL. Liver biopsy revealed hepatosteatosis. She was diagnosed with monogenic diabetes and Nonalcoholic Steatohepatitis (NASH). Patient was given glibenclamide 5 mg twice daily; insulin detemir 14 IU; metformin 500 mg twice daily, with uncooked corn starch. After three months of treatment random blood glucose became 132 mg/dL and A1C became 7.7%; insulin was stopped. Seven months later random blood glucose increased to 287.5 mg/dL, ALT: 204 U/L, and AST: 257 U/L. Insulin was readminestered and glibenclamide were increased to three times daily. A1C evaluation revealed 5.7%. Diabetic nephropathy (DN) occurred, but after a month of captopril, proteinuria was improved from 2.8 g/24 hrs to 1.5 g/24 hrs. Diet for DN was put to therapy. No retinopathy was found. Measurement of quality of life using Diabetes Quality of life (DQOL) revealed satisfaction with life 65.8%, impact of diabetes 55%, worries about diabetes 50.9%, and overall her health was poor. Conclusion is hyperglycemia, nonalcoholic steatohepatitis, hypertriglycemia, and diabetic nephropathy reported as clinical course of monogenic diabetes. The quality of life revealed satisfaction.

